# Characterisation of Mesothelioma-Initiating Cells and Their Susceptibility to Anti-Cancer Agents

**DOI:** 10.1371/journal.pone.0119549

**Published:** 2015-05-01

**Authors:** Elham Alizadeh Pasdar, Michael Smits, Michael Stapelberg, Martina Bajzikova, Marina Stantic, Jacob Goodwin, Bing Yan, Jan Stursa, Jaromira Kovarova, Karishma Sachaphibulkij, Ayenachew Bezawork-Geleta, Margaryta Sobol, Anatoly Filimonenko, Marco Tomasetti, Renata Zobalova, Pavel Hozak, Lan-Feng Dong, Jiri Neuzil

**Affiliations:** 1 School of Medical Science, Griffith University, Southport, Queensland, Australia; 2 Institute of Biotechnology, Academy of Sciences of the Czech Republic, Prague, Czech Republic; 3 Biomedical Research Center, University Hospital Hradec Kralove, Hradec Kralove, Czech Republic; 4 Institute of Molecular Genetics, Academy of Sciences of the Czech Republic, Prague, Czech Republic; 5 Department of Molecular and Clinical Sciences, Polytechnic University of Marche, Ancona, Italy; University of Pennsylvania School of Medicine, UNITED STATES

## Abstract

Malignant mesothelioma (MM) is an aggressive type of tumour causing high mortality. One reason for this paradigm may be the existence of a subpopulation of tumour-initiating cells (TICs) that endow MM with drug resistance and recurrence. The objective of this study was to identify and characterise a TIC subpopulation in MM cells, using spheroid cultures, mesospheres, as a model of MM TICs. Mesospheres, typified by the stemness markers CD24, ABCG2 and OCT4, initiated tumours in immunodeficient mice more efficiently than adherent cells. CD24 knock-down cells lost the sphere-forming capacity and featured lower tumorigenicity. Upon serial transplantation, mesospheres were gradually more efficiently tumrigenic with increased level of stem cell markers. We also show that mesospheres feature mitochondrial and metabolic properties similar to those of normal and cancer stem cells. Finally, we show that mesothelioma-initiating cells are highly susceptible to mitochondrially targeted vitamin E succinate. This study documents that mesospheres can be used as a plausible model of mesothelioma-initiating cells and that they can be utilised in the search for efficient agents against MM.

## Introduction

Malignant mesothelioma (MM), the primary tumour of the pleura, is highly aggressive, with very little if any therapeutic options. Typically being diagnosed 20 to 40 years after exposure to asbestos, the main carcinogen of MM, this neoplastic disease is very aggressive and the mortality rate is exceedingly high with a few months survival after diagnosis [1, 2], the relapse of the tumour occurring shortly after the initiation of treatment [3]. Despite the current ban of asbestos use in industrialised countries and due to the lack of restrictive legislation on the processing and use of asbestos in developing countries [4], MM incidence continues to rise as a result of the long latency period.

It has been suggested that tumour heterogeneity, as a consequence of genetic instability and niche factors within the tumour, is a major cause of resistance to treatment in cancer patients [5, 6]. Genomic studies of MM tumours also highlight inter- and intra-tumour heterogeneity of this type of malignancy [7, 8]. The underlying mechanisms of tumour heterogeneity are still under intense debate and different models have been suggested to define this phenomenon [9]. The proposed cancer stem cell (CSC) model can plausibly describe the heterogeneity and hierarchical organisation of cells within tumours [10]. CSCs (also referred to as tumour-initiating cells, TICs), a small sub-population of cells within carcinomas, have the ability to self-renew and generate differentiated cells with high proliferative capacity that (re-)form the tumour mass [11], as well as endowing tumours resistant to treatment [12, 13]. The presence of TICs may also, in part, explain high resistance of MM to therapy, although this aspect of MM pathophysiology is only partially understood [14, 15].

In this communication, we report on the existence of TICs in mesothelioma by characterising spheres derived from different mesothelioma cell lines as a previously established model for culturing stem cells and TICs [16–18]. We also present characterisation of MM TICs and their susceptibility to anti-cancer agents.

## Materials and Methods

### Cell culture

The established human MM cell lines Ist-Mes-2 (epithelioid histotype), Meso-2 (sarcomatoid histotype), MM-BI (biphasic histotype) [19], and the murine AE17 cell line (epithelioid histotype) [20] were cultured in DMEM supplemented with 10% FBS and the antimycotic/antibiotic cocktail (Invitrogen). The cells were incubated at 37°C in a humidified atmosphere of 5% CO_2_. For sphere formation, adherent cells were cultured at the density of 10^4^ to 2x10^4^ cells/ml of serum free medium (SFM) comprising DMEM-F12 medium (Invitrogen) supplemented with the mouse NeuroCult Proliferation Supplement (Stemcell Technologies), 20 ng/ml human recombinant EGF and 20 ng/ml FGF2 (R&D Systems) at 37°C and 5% CO_2_. Under these conditions cells grow in non-adherent spherical clusters (mesospheres). Proliferation assays were performed using the standard crystal violet method.

### Limited dilution assay

Adherent cells and mesospheres were dissociated and different number of cell from 100 to 0.25 cells per well placed in 96-well cell culture plates containing 200 μl SFM. The cells were incubated at 37°C and 5% CO_2_ for 7 days and their ability to form at least one sphere was evaluated based on a published method [21, 22].

### Tumour cell implantation

Ist-Mes-2 adherent cells and mesospheres were collected, washed with PBS and suspended in 125 μl of serum free-DMEM/Matrigel (BD Bioscience) mixture (1:1, v/v) followed by subcutaneous injection into the right or left mid-abdominal area of Balb-c/nude or NOD/SCID mice using a 23-gauge needle. Tumour formation and progression was monitored and quantified using the Vevo770 USI device equipped with the RMV708 scan-head (VisualSonics) operating at the frequency of 80 MHz and with the resolution of 30 μm. Animal studies were done according to the guidelines of the Australian and New Zealand Council for the Care and Use of Animals in Research and Teaching and were approved by the Griffith University Animal Ethics Committee (permit number MSC/13/10/AEC). All surgery was performed under isoflurane anesthesia, and all efforts were made to minimise suffering.

### Immunohistochemistry, confocal microscopy and TEM

Mice were sacrificed, and their tumours were immediately removed, fixed in 4% phosphate-buffered formaldehyde and embedded in paraffin. Tumours were sectioned at 7 μm thickness on a Leica RM2265 Microtome (Leica Microsystems), mounted on Supersoft Plus glass slides (Lomb Scientific), dried overnight at 37°C, de-parafinised in xylene and rehydrated according to the routine histopathological procedure, and stained with H&E. For confocal microscopy, the processed slides were incubated overnight with anti-mesothelin IgG (Abcam) followed by HRP-conjugated secondary IgG and imaged using the Olympus FV1000 fluorescence confocal microscope. TEM of sphere cells was performed using an established protocol.

### Western blot analysis

The cells were harvested and lysed with the lysis buffer (Cell Signalling). Samples were resolved on SDS-PAGE gels and transferred onto PVDF membranes, which were blocked with 5% BSA, and incubated with primary and HRP-conjugated secondary IgGs, followed by visualisation using the ChemiDoc XRS^+^ System (Biorad). Primary antibodies were as follows: anti-CD24 IgG, anti-EpCAM IgG (Abcam), anti-CD47 IgG (Santa Cruz), anti-Oct3/4 IgG (Stem Cell Technologies), anti-ABCG2 IgG, anti-phospho-p44/42 MAPK (pERK1/2) (Thr202/Thr204) IgG, anti-p44/p42 (ERK1/2) IgG, anti-P38 MAPK IgG, anti-phospho-P38 MAPK (pP38) (Thr180/Thr182) IgG, anti-EGFR IgG, anti-phospho-EGFR (pEGFR) (Tyr1068) IgG (all Cell Signaling). Anti-actin IgG (Sigma) was used as a loading control.

### Quantitative real-time polymerase chain reaction (qPCR)

Total mRNA was extracted using the RNeasy Kit (Qiagen) and cDNA synthetised by means of the RevertAid First Strand cDNA Synthesis Kit (Thermo Scientific) and iCycler iQ Real-Time PCR Detection System. qPCR was performed using the Platinum SYBR Green qPCR SuperMix-UDG (Invitrogen). Generally, 200 ng of template total RNA and 0.2 μM of gene-specific PCR primers were used per reaction, and the Eco Real-Time PCR System instrument (Illumina) for amplification. GAPDH was used for normalization, the relative levels of target genes were quantified using the ΔΔCt method. Individual primers are as follows. CD24: forward, AGC GGT TCT CCA AGC ACC CA, reverse—TAG GAG CAG TGC CAG CAG CA; OCT4: forward—CCC CAG GTT GGA GTG GGG CT, reverse—GGA GGC CCC ATC GGA GTT GC; ABCG2: forward—ACG AAC GGA TTA ACA GGG TCA, reverse—CTC CAG ACA CAC CAC GGA T; SOX4: forward GAT TCC GCC CAC AGA GAG TC, reverse—GGT AGC TCA GGA AAG CGA CA; KLF4: CTG GGT CTT GAG GAA GTG CT, reverse—GGC ATG AGC TCT TGG TAA TGG; c-Myc: forward—GGA CTT GTT GCG GAA ACG AC, reverse—CTC AGC CAA GGT TGT GAG GT; ABCB5: forward—ACT GAG AAA GGA AGC AGT TGG A, reverse—TAA AGG AAG GCA GGC TCC AT; GAPDH: forward—TGC ACC ACC AAC TGC TTA GC, reverse—GGC ATG GAC TGT GGT CAT GAG.

### Transfection with small hairpin RNA (shRNA)

Human CD24-specific shRNA constructs in the pRS backbone under control of the U6 promoter and the relevant empty vector were obtained from OriGene Technologies (Rockville). The FuGENE HD transfection reagent (Promega) was used to stably transfect Ist-Mes-2 cells with the shRNA CD24 and empty vector (mock-transfected cells) according to the manufacturer’s instructions. The stable single cell-derived clones were selected by the addition of 4 μg/ml of puromycin to the culture medium and monitored for transfection using qPCR or western blotting.

### Assessment of superoxide generation, mitochondrial membrane potential, mitochondrial mass, cell cycle, apoptosis and cell size

Intracellular superoxide anion levels were evaluated using the fluorescent probe MitoSOX. 10^5^ cells were cultured in 24-well plates and at 70% confluency, were treated with 20 μM MitoSOX for 30 min, washed re-suspended in PBS, and analysed by flow cytometry (FACS Fortessa, Becton Dickinson). Superoxide levels were expressed as mean fluorescence intensity (MFI). Mitochondrial mass was evaluated by treating the cells with 50 μM MitoTracker Green FM for 30 min and analysing by flow cytometry. For assessment of cell cycle, 10^6^ viable cells were washed and fixed in 70% EtOH overnight and stained with 100 μg/ml propidium iodide (PI), and analysed by a FACS Calibur flow cytometer (Becton-Dickinson). Signals emitted from a single nucleus and aggregated cells were excluded using a double-gating module. Apoptosis was evaluated by the standard annexin V/PI method. Cell size was estimated using the Scepter handheld automated cell counter (Millipore) according to manufacturer's instruction.

### CS, SDH and SQR activity

CS activity was evaluated by reduction of DTNB [5,5′-dithiobis-(2-nitrobenzoic acid)] by the enzyme and its coupled reaction with oxaloacetate and acetyl-CoA. Briefly, 10 μg of protein lysate was added to a mixture of 10 mM DTNB and 30 mM acetyl coenzyme A. The change in absorbance of DTNB was measured in the Tecan Infinite 200 PRO reader for 90 s at 412 nm after the addition of 10 mM oxaloacetate in a 200 μl reaction volume. SDH activity was assessed as previously described (Dong et al., 2011). For SQR activity, 40 μg of protein lysate was added to 1 ml of the SQR assay buffer (10 mM KH2PO4, pH 7.8, 2 mM EDTA, 1 mg/ml BSA), 80 μM dichlorophenolindophenol, 0.2 mM ATP, 4 μM rotenone and 10 mM succinate, and incubated at 30°C for 10 min. The absorbance was measured every minute for 30 min after initiating the reaction by decylubiquinone (80 μM) [23].

### High-resolution respirometry

Oxygen consumption was assessed using the Oxygraph-2k high-resolution respirometer and the data analysed using the Datlab software (both Oroboros), essentially as described [24]. All measurements were performed at 37°C in 2 ml chambers. For evaluation of intact cells, serum-free DMEM media was used, while digitonin-permeabilised cells were suspended in the MiR05 mitochondrial respiration medium. For all experiments, 10^6^ cells were used per chamber. The rate of oxygen consumption (oxygen flux) was calculated as the negative slope of oxygen concentration. Cellular oxygen flux was corrected for the instrumental background, which results from the non-specific sensor oxygen consumption. The conditions for respiration were as follows: GM_L—glutamate (2 mM)/malate (10 mM); GM_P—as before plus ADP (2.5 mM); GMS_P—as before plus succinate (10 mM); GMS_E—as before plus CCCP (5 μM); S(Rot)_E—as before plus rotenone (0.5 μM). The symbols stand for: GM_L—leak (oxygen consumption not coupled to ATP production); GM_P—respiration via CI; GMS_P—respiration via CI+CII; GMS_E—uncoupled respiration; S(Rot)_E—uncoupled respiration via CII.

### Glucose uptake, ATP level and lactate assays

For glucose uptake, cells were incubated in low glucose (1 g/l) DMEM overnight at 5% CO_2_ and 37°C. They were then incubated for 30 min with 50 μM 2-nitrobenzodeoxyglucose (2-NBDG) in low-glucose DMEM. The level of fluorescent glucose analogue in the cells was evaluated by flow cytometry and expressed as MFI. For ATP estimation, cells were seeded in 96-well plates at 10^4^ cell per well and intracellular ATP levels analysed using a luciferase-based kit (CellTiter-Glo Luminescent Assay, Promega). The results were normalised to total protein. For lactate assay, cells were seeded at 10^4^ per well in 96-well plates. After 24 h incubation, the cell culture medium was used for the evaluation of lactate using a fluorometric kit (Cayman Chemicals). The results were normalised to total protein.

### Statistical analysis

The paired Student's t-test was used to assess the differences between experimental results, with differences with *p*<0.05 considered as statistically significant.

## Results

### Formation of mesospheres from MM cell lines with various phenotypes

**Fig 1:**
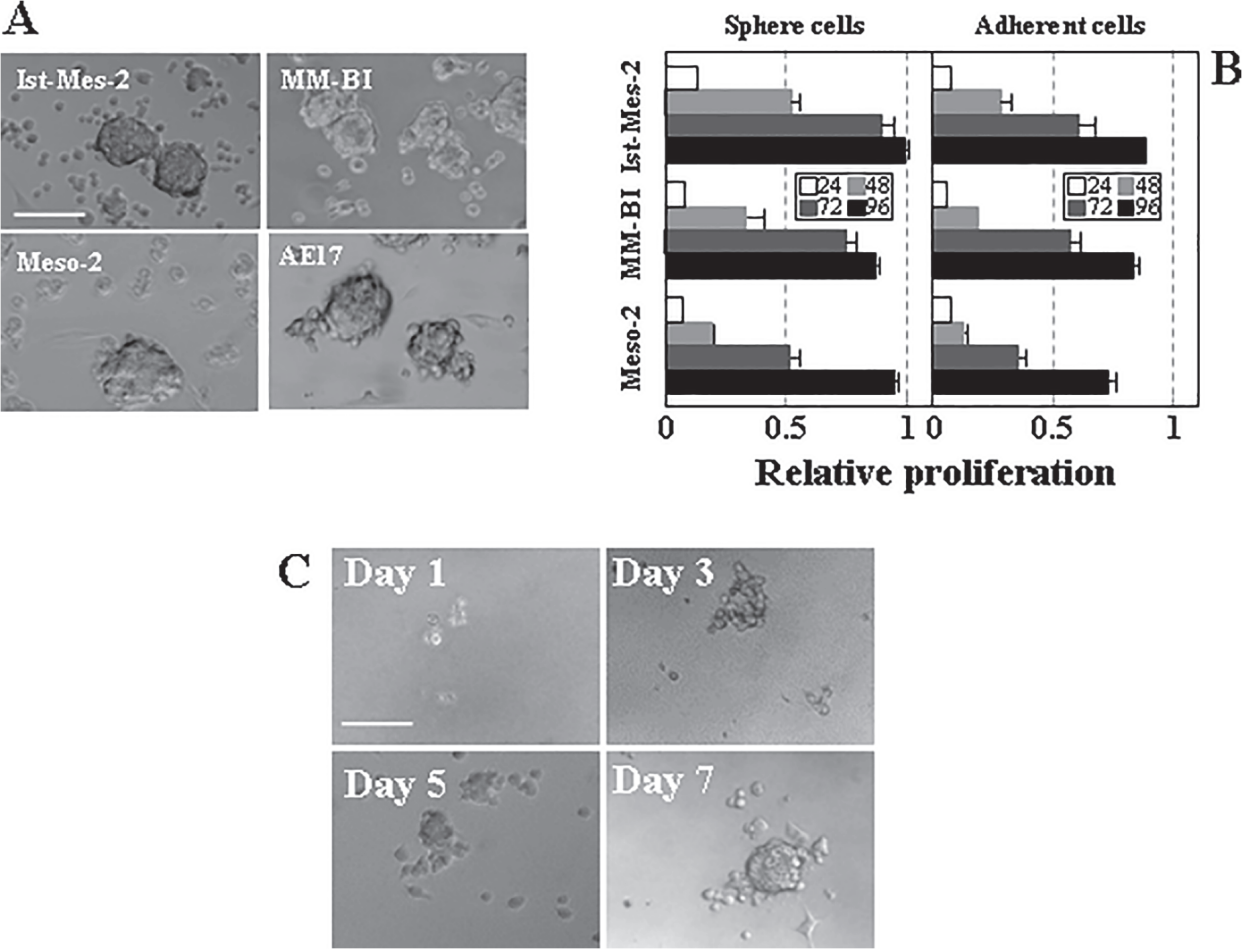
Mesothelioma cells form spheres with different intensity. (A) Phase contrast images of mesospheres from established MM cell lines. (B) Proliferation rate of single cell-derived spheres re-cultured in normal differentiation medium compared to parental mesothelioma cells. For this experiment, cells were seeded at the same number into adherent medium, and proliferation of the initially adherent and sphere cells as evaluated using the MTT assay. The ratio of proliferation rate in sphere-derived adherent cells incubated for 72 h is 3.37 for Ist-Mes-2, 1.72 for Meso-2 and 4.25 for MM-BI cells. (C) A single Ist-Mes-2 cell was maintained in SFM and sphere formation on days 1, 3, 5 and 7 documented. The data in panel B are mean values ± S.D. derived from three independent experiments. Images in panels A and C are representative of three individual experiments. The scale bar in panels A and C represents 100 μm.

A major characteristic of TICs is their ability to self-renew via asymmetrical cell division. To verify that MM cells comprise a sub-population with distinctive self-renewal capacity, established MM cell lines including Ist-Mes-2 (epithelial histotype), Meso-2 (sarcomatoid histotype) and MM-BI cells (biphasic histotype), as well as murine AE17 MM cells were cultured under conditions established for enriching cell populations in TICs. For the enrichment in TICs, cells were cultivated in serum-free medium (SFM) [25], a condition maintaining cells in an undifferentiated state that has been previously used to isolate various types of stem cells and TICs [26]. In SFM, all MM cells grew in spherical colonies, referred to as mesospheres, with the diameter of 30–100 μm after 3–5 days in culture (Fig 1A). For all MM cell lines studied, adherent cells proliferated less efficiently than the corresponding sphere cells when placed in complete medium, and Ist-Mes-2 cells were more proliferative than MM-BI and Meso-2 cells (Fig 1B). Mesospheres maintained the ability to form new spheres after mechanical dissociation and re-seeding into fresh SFM. The frequency of sphere-forming cells representing TICs was evaluated by the limiting dilution assay as previously described [16, 21, 22]. It can be calculated from the plots, based on the intercept at the 37% value with the slopes of the individual cell lines that the number of cells required for the formation of one sphere was 1.81±0.24 for Ist-Mes-2, 4.54±0.39 for MM-BI and 7.53±0.89 for Meso-2 cells (Table 1). That a single cell can form a sphere when placed to SFM is documented in Fig 1C, showing time-dependent sphere formation starting with a single Ist-Mes-2 cell.

**Table 1:** Number of cells needed to form a sphere.

Cell line	Number of cells
Ist-Mes-2	1.8±0.2^a^
MM-BI	4.5±0.4
Meso-2	7.5±0.9

^a^The number of cells needed to form a sphere was assessed as described [21, 22].

### Mesospheres express stem cell markers and efficiently generate tumours

**Fig 2:**
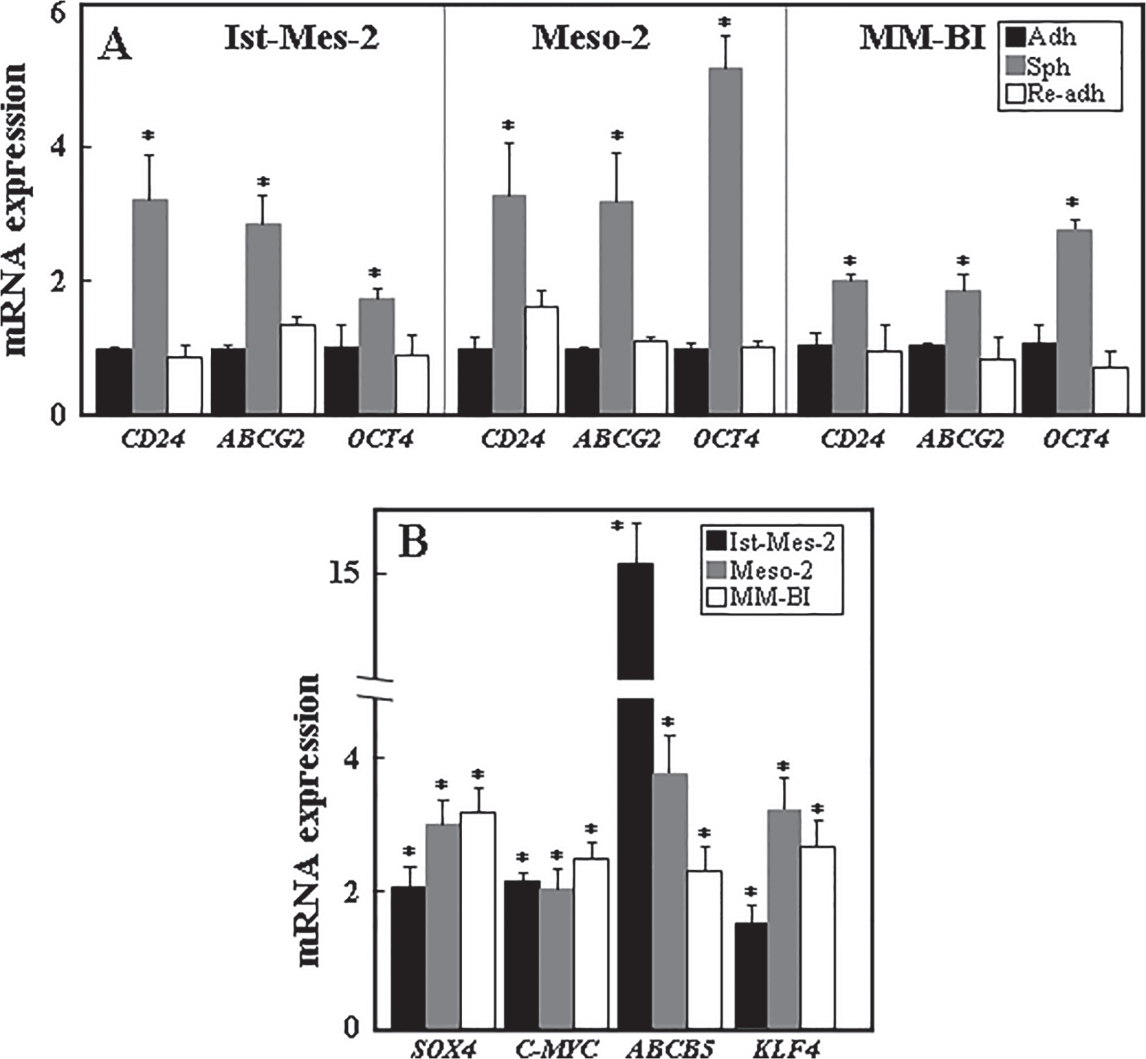
Sphere cells derived from MM cell lines show increased levels of ‘stemness’ markers. (A) Ist-Mes-2, Meso-2 and MM-BI cells were allowed to form spheres, after which these were placed in the complete medium to cause differentiation and re-adhesion of the cells. Relative levels *CD24*, *ABCG2* and *OCT4* mRNAs were assessed in the original adherent cells (set as 1), the spheres and the re-adherent cells. (B) Ist-Mes-2, Meso-2 and MM-BI cells spheres were tested for the level of *SOX4*, *C-MYC*, *ABCB5* and *KLF4* mRNA and their level expressed relative to that of adherent cells. The data are mean values ± S.D. derived from three independent experiments, the symbol ‘*’ stands significantly different data with *p*<0.05, ‘**’ for those with *p*<0.005.

We have recently performed global gene expression analysis of sphere and adherent Ist-Mes-2 cells that documented a ‘stemness signature’ of Ist-Mes-2 mesospheres [27]. To verify the results of microarray data and to further establish the characteristics of the sphere cells, we analysed both spheres and adherent monolayer cells for the expression of markers that are often associate with TICs [10] and that were also found increased in the microarray analysis of Ist-Mes-2 spheres [27]. Higher expression of *CD24*, *OCT4* and *ABCG2* mRNA (1.5 to 4-fold increase) was found in spheres compared to corresponding adherent cells. To test for plasticity of MM cells, single cell-derived spheres were subjected to normal adherent culture conditions after forming mesospheres to allow their re-attachment. The results indicate that upon differentiation, adherent cells derived from spheres show similar level of expression of stem cell markers to that in the original adherent cells (Fig 2A). Mesospheres also showed increased expression of several other markers of stemness, i.e. *SOX4*, *C-MYC*, *ABCB5* and *KLF4* (Fig 2B).

**Fig 3:**
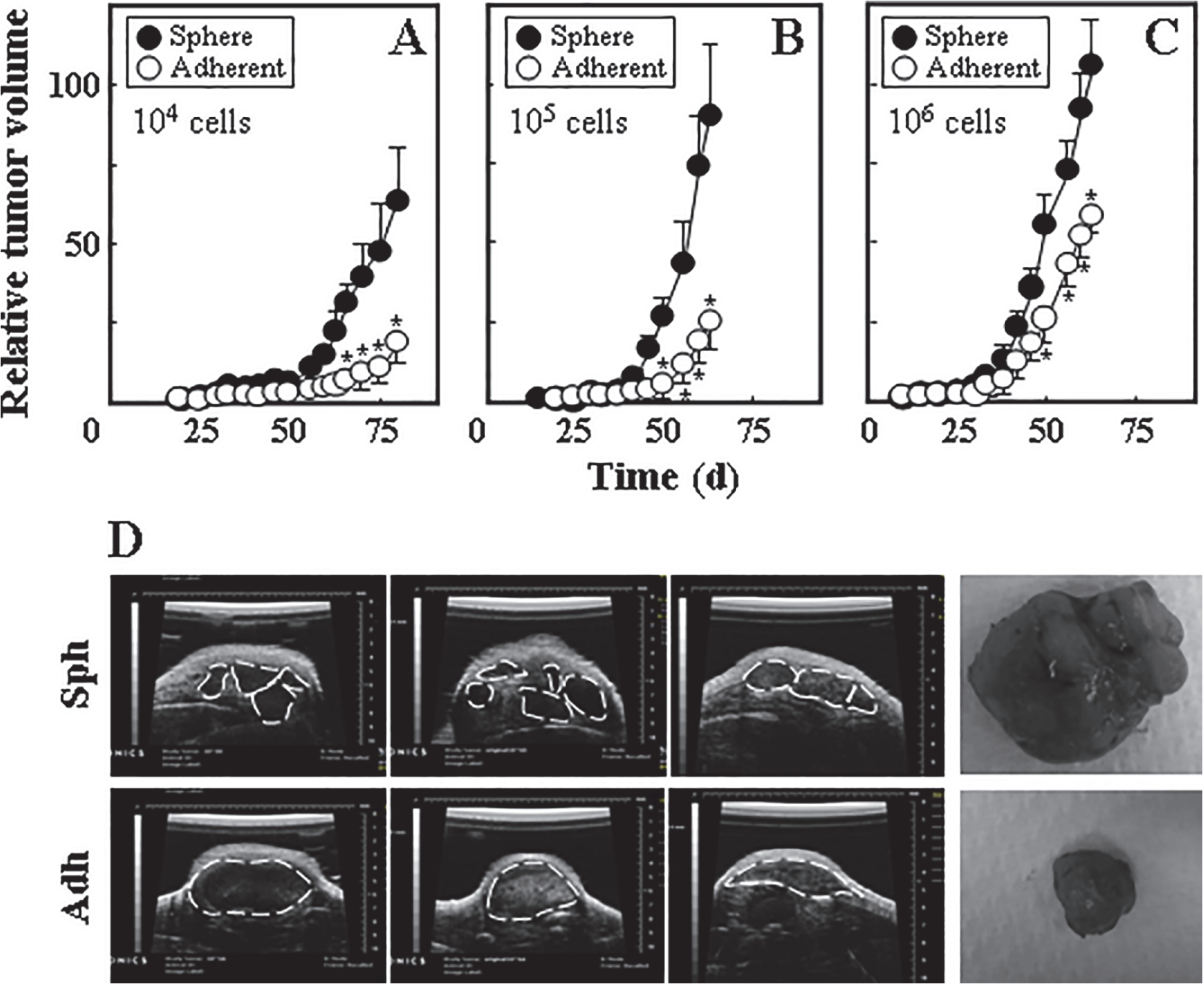
Ist-Mes-2 sphere cells form tumours more efficiently than adherent cells. Ist-Mes-2 adherent and sphere cells were injected s.c. into Balb-c/nude mice at 10^4^ (A), 10^5^ (B) and 10^6^ cells per mouse (C). Tumour volume was evaluated using USI and is expressed relative to the initially detected tumour (~20 mm^3^). (D) Three different representative images taken by ultrasound from sphere- and adherent cell-derived tumours are shown for tumours grown from 10^6^ cells per mouse. The broken line indicates contours of the USI-visualised tumours. On the right hand side, a photograph of a representative tumour grown from 10^6^ sphere (upper image) and adherent cells (lower image) is shown. Data in panels A-C are derived from experiments comprising 4–5 mice per group and are mean values ± S.E.M. The symbol ‘*’ indicates significantly different values with *p*<0.05.

To investigate whether mesospheres and adherent monolayer cells differ in their ability to form tumours in immunocompromised mice, Ist-Mes-2 mesosphere and adherent cells were injected subcutaneously into Balb-c/nude mice at 10^4^, 10^5^ and 10^6^ cells per mouse, and tumour initiation and progression were monitored by ultrasound imaging (USI). Sphere-derived cells were more efficient in tumour formation than their adherent counterparts, in particular for the lower cell numbers (Fig 3A–3C). Inspection of tumour images acquired by USI revealed multilobular nature of sphere-derived tumours, which is not obvious in the adherent cell-derived carcinomas (Fig 3D).

### Mesospheres propagate serial tumours with increasingly aggressive nature

**Fig 4:**
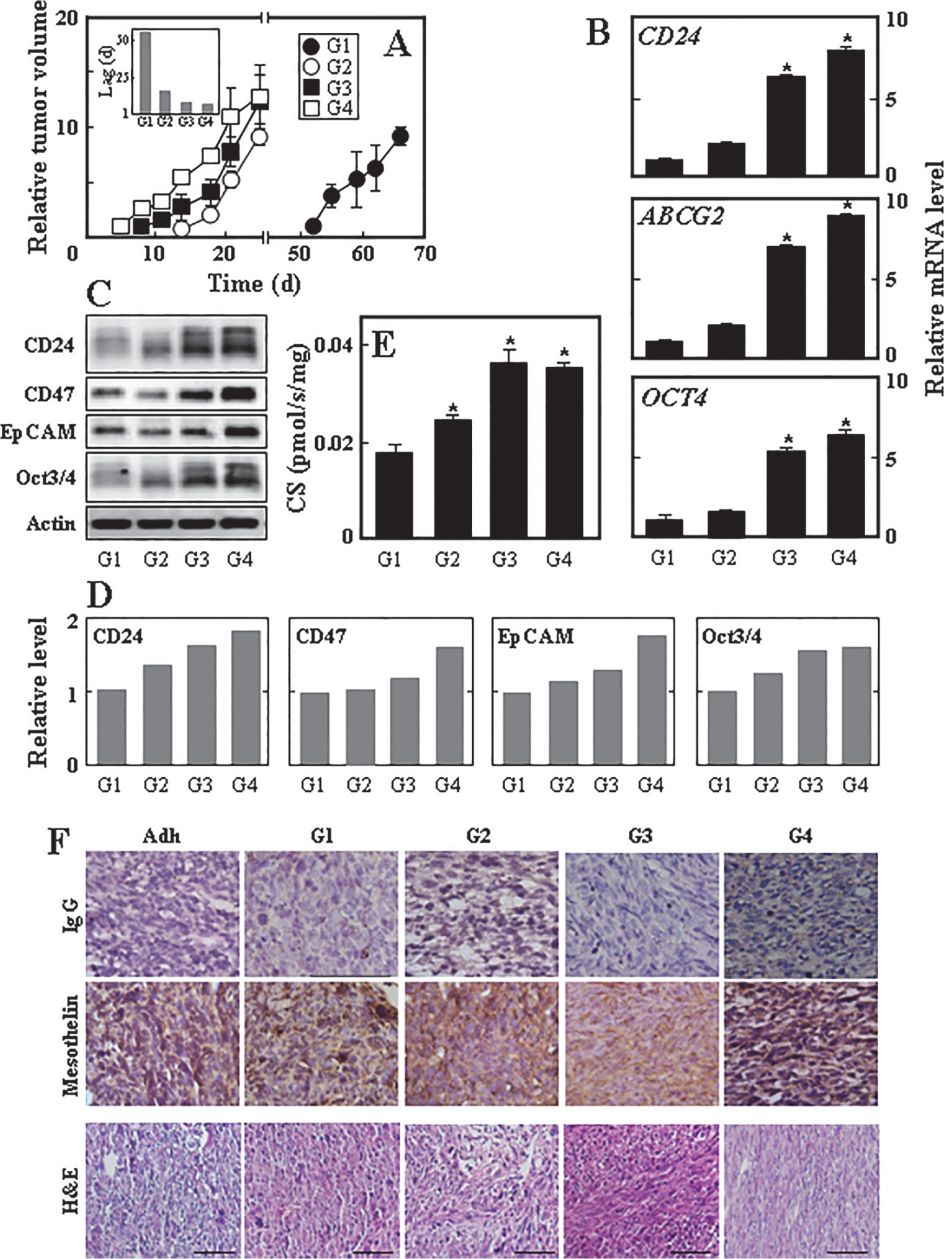
Mesospheres form tumours in serial transplantations. (A) Ist-Mes-2 sphere cells (generation 1, G1) were subcutaneously grafted into Balb-c/nude mice at 10^6^ cells per animal. When tumours reached about 2,000 mm^3^, the mice were sacrificed, tumours excised and malignant cells grew *in vitro* as a cell line. The adherent cells were converted into spheres (G1) and these were grafted into Balb-c/nude mice to form tumours that were used for generation 2 (G2) spheres. This procedure was repeated two more times to derive G3 and G4 spheres. The inset in panel A shows the lag to tumour appearance following cell grafting for individual sphere cell generations. Cells of individual generations were evaluated for stemness markers as shown using qPCR (B) and WB (panel C shows the blots, panel D their densitometric evaluations) and for CS activity (E). (F) Tumours derived from adherent cells and spheres of individual generations were excised, paraffin-embedded and stained for the MM marker mesothelin (upper images show staining with the exclusion of the primary IgG) and with H&E. Data shown in panel A are derived from 5 animals and are mean values ± S.E.M, data in panels B and E are mean values from three independent experiments ± S.D. Images in panel C are representative of two individual experiments and their densitometric evaluations in panel D represent mean values with differences lower than 10%. Images in panel F were obtained using one tumour for each condition (generation). The symbol ‘*’ denotes statistically significant differences with *p*<0.05. Images are representative of three different tumours.

To verify whether mesosphere cells can form tumours sequentially due to the self-renewal capacity of TICs, we subjected them to serial xenografting into Balb-c/nude mice. Ist-Mes-2 sphere cells were allowed to form a tumour, and MM cells derived from the tumour were then placed in culture resulting in a cell line (1^st^ generation, G1). These cells were used again to form spheres that were grafted in nude mice to give second generation of tumours from which 2^nd^ generation cell line (G2) was derived. This procedure was repeated twice more to obtain G3 and G4 cell lines. With each generation the lag time to tumour initiation shortened such that it was 52 days for G1, 15 days for G2, 7 days for G3 and 5 days for G4 (Fig 4A). Interestingly, we found that the levels of the stem cell marker (CD24, ABCG2, OCT4) mRNAs gradually increased in the individual sphere generations (Fig 4B). We also observed generation-dependent increase in CD24, CD47, EpCAM and Oct3/4 protein, further supporting the notion that stemness increases with each generation (Fig 4C and 4D). To test the mitochondrial function in individual generation, we studied the activity of CS, a component of the tricarboxylic acid (TCA) cycle and a surrogate measure of mitochondrial mass. Fig 4E shows a gradual increase in the activity of the enzyme. To document cancer morphology of tumours derived from each sphere generation, we stained tumour sections for the presence of a marker of MM, mesothelin, and with H&E (Fig 4F).

**Fig 5:**
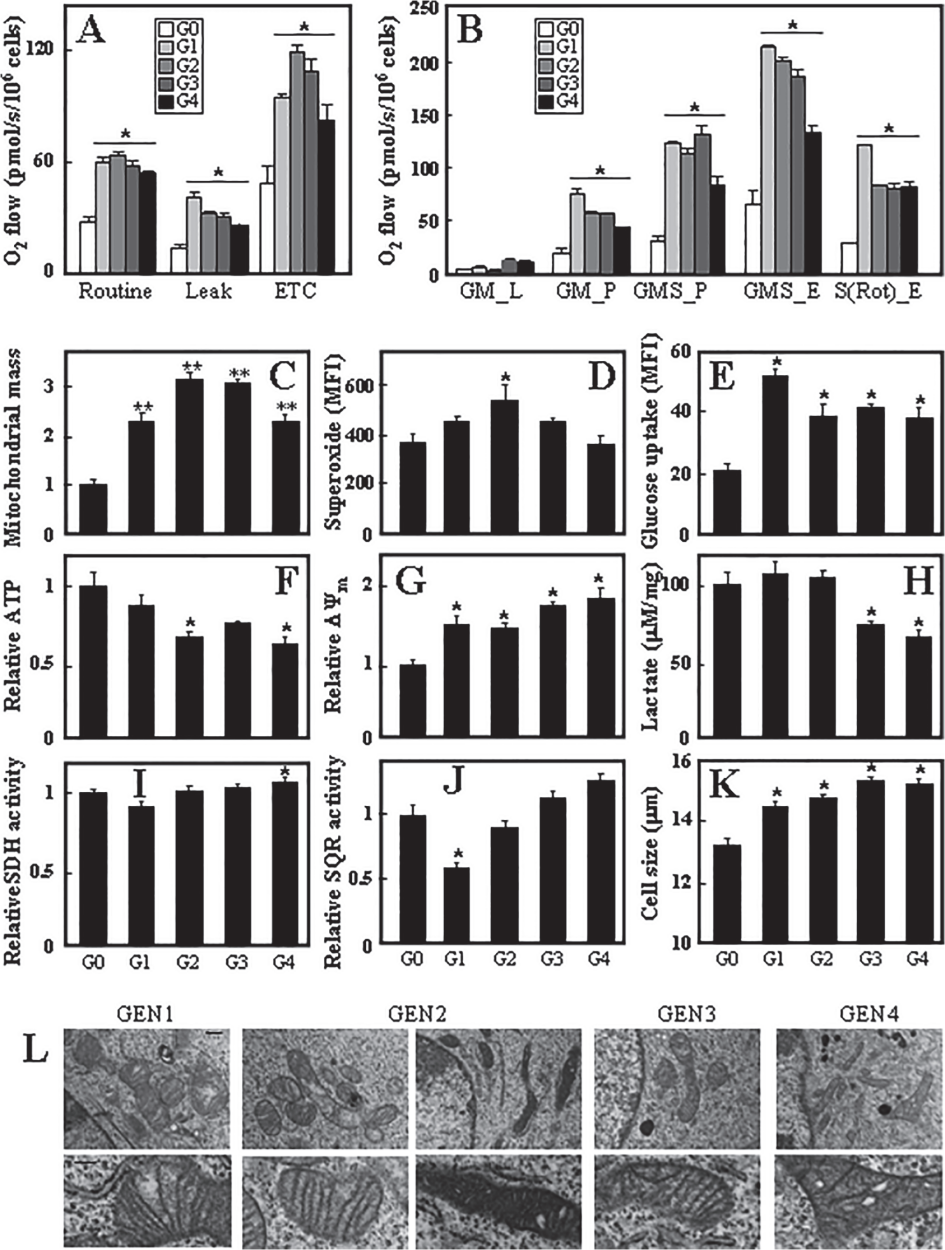
Mesospheres derived from different tumour generations show different mitochondrial features. Sphere cells representing individual generations were evaluated for respiration using the routine protocol (A) and the protocol for permeabilised cells (B) by means of the Oxygraph 2k high resolution respirometer. The symbols GM_L, GM_P, GMS_P, GMS_E and S(Rot)_E represent routine respiration, respiration via complex I, respiration via complex I+II, uncoupled respiration and uncoupled respiration via CII, respectively. (C) Mitochondrial mass was evaluated using MitoTracker Green as detailed in the Methods section. Superoxide was evaluated using the fluorescent probe MitoSOX (D), glucose uptake using the fluorescent glucose analogue 2-NBDG (E), ATP levels by a luciferase-based assay (F), ΔΨ_m_ using the fluorescent probe TMRM (G), lactate levels using a commercial kit (H), for SDH (I) and SQR (J) activities using enzymatic assays, and hand-held cells counter for cell size (K). (L) TEM was performed on individual generation sphere cells as detailed in the Methods section. Data in panels A-K are mean values ±S.D., and are derived from three individual experiments. The symbol ‘*’ denotes statistically significant differences with *p*<0.05. Images in panel L are representative of three independent experiments. The white bar in panel K in the upper images represents 500 nm, in the lower images 200 nm.

Since sphere cultures derived from different generation tumours showed increasing CS activity, we assessed these cells for their mitochondrial function in more detail. We first tested the individual generation sphere cells for respiration. Analysis of the respiration of intact as well as permeabilised cells using the Oxygraph high resolution respirometer revealed that G1 generation cells respired more (both via routine respiration as well as via respiration using complex I, CI, and CII) than G0 cells (spheres prepared from the original adherent Ist-Mes-2 cells). Respiration in G2-G4 cells slightly declined but was still much higher than in the parental sphere cells (Fig 5A and 5B). This trend was quite similar for the mitochondrial mass (Fig 5C), superoxide generation (Fig 5D), glucose uptake (Fig 5E) and mitochondrial potential (Fig 5G). On the other hand, the level of ATP dropped in the individual sphere generations (Fig 5F) and a similar trend was found for lactate production (Fig 5H). We also tested the two activities of CII, the SDH and succinate quinone reductase (SQR) activity. Fig 5I documents no change in SDH and Fig 5J a drop in G1 cells of SQR activity. We observed a slight gradual increase in the size of cells of different generations (Fig 5K). Transmission electron microscopy (TEM) images revealed morphological differences in mitochondria of individual generation cells (Fig 5L). First generation cells feature round or oval shaped mitochondria with cristae arranged parallel to each other and some mitochondria surrounded with rough endoplasmatic reticulum. G2 cells have two types of mitochondria with equal frequency: mitochondria with loosened matrix, and mitochondria with dense matrix and dilated cristae as well as partial localisation of rough ER in the vicinity of mitochondria, similar to G1 cells. G3 cells show dense mitochondria with lamellar but less straight cristae, and G4 cells feature branched mitochondria with dilated (vesicular) cristae and dense matrix.

### CD24 knock-down attenuates the ability of mesothelioma cells to form spheres and form tumours

CD24 is a glycosyl phosphatidyl inositol-linked mucin-like cell surface protein with a role in cell adhesion, and is expressed in various cancers [28–30]. This marker is associated with poor prognosis in various tumours and its down-regulation inhibits proliferation and induces apoptosis in tumour cells [31], whereas increased expression of CD24 promotes tumour growth and metastasis [32].

**Fig 6:**
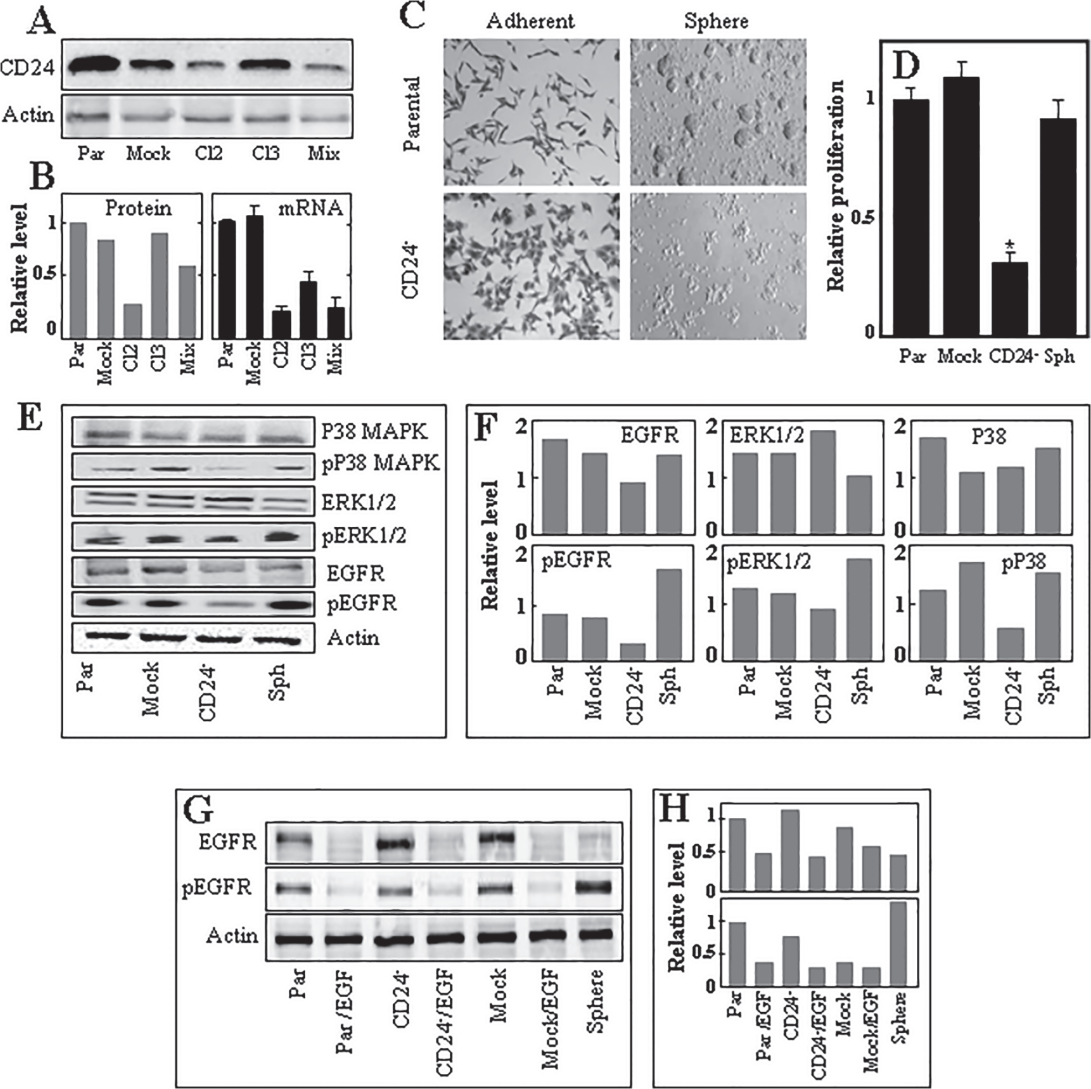
CD24 is important for mesosphere formation and MM cell proliferation. (A) Parental, mock-transfected, clone 2, clone3 and ‘mixed’ cells (Ist-Mes-2 cells transfected with CD24 shRNA and selected for several clones) were assessed for CD24 using western blotting with actin as the loading control. Relative level of CD24 in the cells is shown based on densitometric evaluation in panel B, which also document CD24 mRNA level related to *GAPDH*. (C) Parental and CD24^-^ Ist-Mes-2 cells (clone 2) were grown either in the serum-containing medium or the SFM. (D) Parental, mock-transfected and CD24^-^ adherent and sphere Ist-Mes-2 cells were assessed for proliferation at 48 h after seeding in culture at identical cell number using the crystal violet assay. (E) Parental, mock-transfected and CD24^-^ adherent and sphere Ist-Mes-2 cells were evaluated for P38, pP38 MAPK, ERK1/2, pERK1/2, EGRF and pEGRF by western blotting with anti-actin IgG as a loading control, with densitometric evaluation of the blots in panel F. (G) Parental, CD24^-^ or mock transfected cells grown either in the complete medium or in complete medium supplemented with recombinant EGF, or sphere cells grown in the SFM were assessed for EGFR and pEGFR by western blotting using anti-actin IgG as a loading control, with densitometric evaluation shown in panel H. Data in panels B (right panel) and D are mean values ±S.D., and are derived from three individual experiments. The symbol ‘*’ denotes statistically significant differences with *p*<0.05. Images in panel C are representative of three independent experiments, and images in panels A, E and G of two individual experiments. The densitographic evaluations in panels B (left), F and H are average values of two evaluations with differences less than 10%.

To further verify the role of CD24, highly expressed in Ist-Mes-2 spheres, in the ‘stemness’ of MM cells, its expression was stably down-regulated using shRNA. We selected several clones of the knock-down cells, of which clone 2 CD24^-^ cells showed about 80% decrease of CD24, as documented both by western blotting and qPCR (Fig 6A and 6B). We then performed all experiments with the clone 2 cells. CD24^-^ Ist-Mes-2 cells acquired epithelial-like morphology and showed a loss of the parental cells’ propensity to form spheres (Fig 6C). More specifically, no sign of sphere formation was observed after maintaining CD24^-^ cells in SFM for 7 days, and the cells continued to persist in the culture for few more days as loose aggregates before they died. CD24 knock-down also significantly suppressed proliferation of Ist-Mes-2 cells, as documented in Fig 6D. We next assessed the role of CD24 in the mitogenic MAPK pathway, that is triggered from the epidermal growth factor receptor (EGFR) and that has been shown to be overexpressed in MM [33]. Our data indicate relatively high level of phosphorylated EGFR in mesospheres, while decreased amounts of pEGFR were observed in CD24^-^ cells. Of the downstream proteins of the MAPK pathway, CD24^-^ cells show lower phosphorylation of P38 but not ERK1/2, while P38 was found phosphorylated in sphere cells (Fig 6E and 6F). Finally, we performed a control experiment to rule out the possibility that phosphorylation of EGFR in spheres is due to the presence of EGF in the media. Adherent parental, CD24^-^ and mock-transfected Ist-Mes-2 cells were maintained in the complete medium for 24 h in the presence or absence of added EGF and, together with sphere cells that were maintained in the EGF-containing SFM, assessed by WB for EGFR and pEGFR (Fig 6G and 6H). The results indicate that the presence of EGF in the medium used for cultivation of adherent cells does not support phosphorylation of EGFR, which is therefore an intrinsic property of the sphere cells.

**Fig 7:**
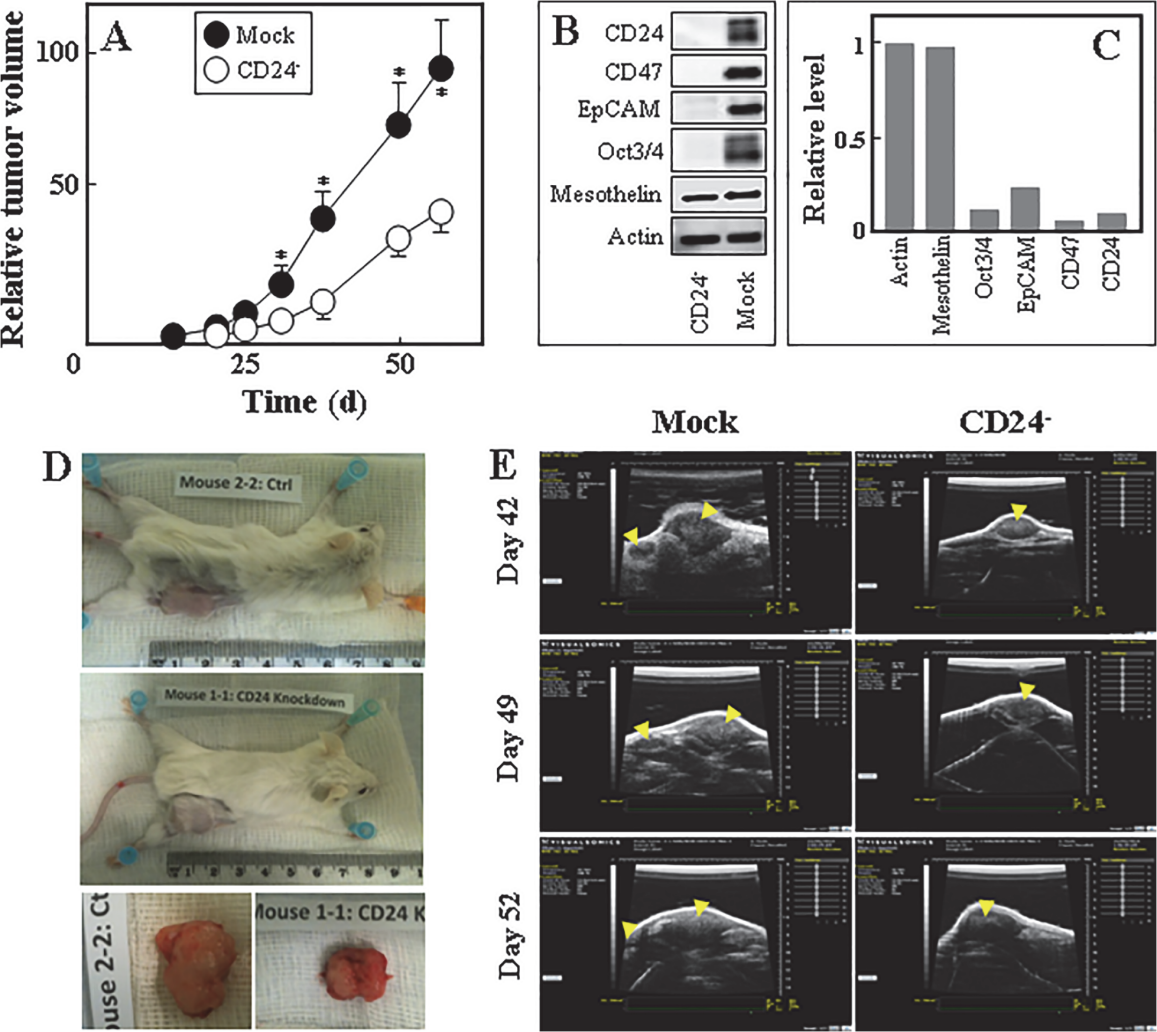
CD24 supports initiation and progression of mesotheliomas. (A) Mock-transfected and CD24^-^ Ist-Mes-2 cells (10^6^ per animal) were grafted subcutaneously into NOD/SCID mice and tumour formation followed using USI. (B) CD24^-^ and mock-transfected Ist-Mes-2 cell-derived tumours were evaluated for CD24, CD47, EpCAM and Oct3/4 by western blotting using anti-actin IgG as a loading control, with panel C documenting densitometric evaluation of the blots. (D) A representative image of a mouse with CD24^-^ cell-derived tumour and parental cell-derived tumour is shown. (E) Representative USI images of a tumour derived from mock-transfected and CD24^-^ cells acquired on different days are shown, with the yellow arrows indicating the position of the tumours. Data in panel A are mean values ±S.E.M., and are derived from four animals. The symbol ‘*’ denotes statistically significant differences with *p*<0.05. Images in panel B are representative of two independent experiments with the densitometric evaluation showing average data with differences less than 10%.

We next tested the effect of CD24 knock-down on the propensity of MM cells to form tumours, such that mock-transfected and CD24^-^ Ist-Mes-2 cells were grafted in NOD/SCID mice and tumour growth kinetics evaluated by USI. Tumours derived from CD24^-^ cells progressed significantly slower than those derived from control (mock-transfected) cells (Fig 7A). Interestingly, we found that tumours from CD24^-^ cells did not express the CD24 protein, and neither did they express the stemness markers CD47, EpCAM and Oct3/4 (Fig 7B and 7C). Tumours derived from control cells were highly aggressive and invaded the dorsal abdominal wall, while the smaller CD24^-^ cell-derived tumours were localised subcutaneously (Fig 7D and 7E). These data highlight the important role of CD24 in the stemness and progression of experimental mesotheliomas.

**Fig 8:**
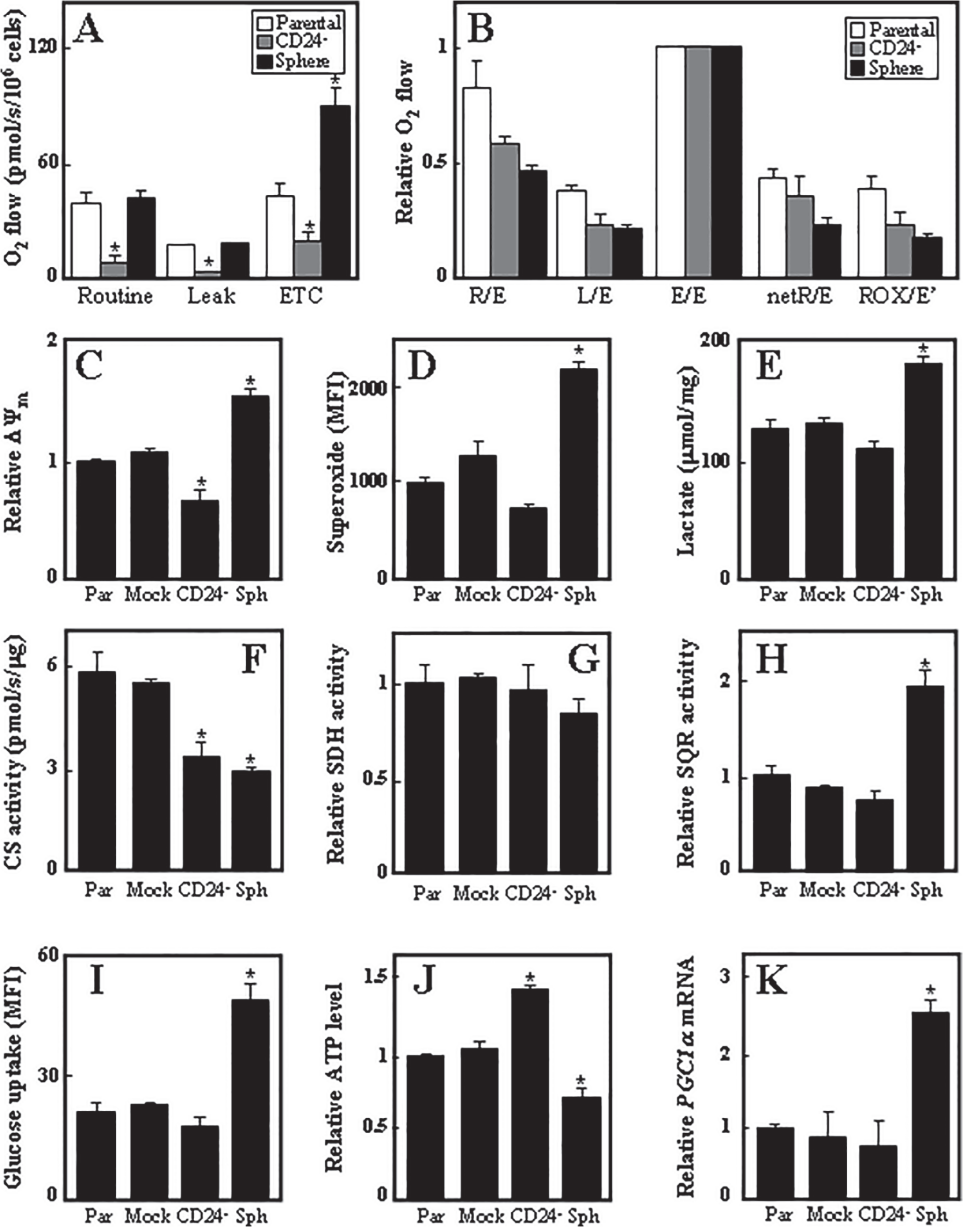
Effect of CD24 knock-down on mitochondrial function. Parental and CD24^-^ adherent and sphere Ist-Mes-2, non-permeabilised cells were evaluated for routine respiration using the Oxygraph instrument (A). Panel B shows respiration related to the maximum respiratory capacity of the cells (ETC). The symbols in panel A stand for: R, routine, L, leak, E, ETC, netR, R-L, ROX, residual respiration. Parental, mock-transfected, CD24^-^ adherent and sphere Ist-Mes-2 cells were evaluated for ΔΨ_m_ using TMRM (C), superoxide generation using MitoSOX (D), lactate production using a commercial kit (E), citrate synthase (CS) activity (F), relative SDH (G) and SQR activity (H), glucose uptake (I) and ATP level (J). Panel K documents the level of *PCG1α*mRNA in parental, mock-transfected and CD24^-^ adherent and sphere Ist-Mes-2 cells. Data shown are mean values ±S.D., and are derived from three individual experiments. The symbol ‘*’ denotes statistically significant differences with *p*<0.05.

Parental and CD24^-^ adherent and sphere Ist-Mes-2 cells were evaluated for various mitochondrial features. Routine respiration as well as the respiratory capacity (ETC) were much lower in CD24^-^ cells compared to parental adherent cells, while sphere cells were found to feature considerably higher respiratory capacity (Fig 8A). Flux control ratios, expressing respiration independent of mitochondrial content, were then calculated, showing that the ratios are lower for CD24^-^ and, in particular, for sphere cells (Fig 8B). ΔΨ_m,i_ was found lower in CD24^-^ cells and higher in sphere cells (Fig 8C), which was also the case for superoxide generation (Fig 8D), glucose uptake (Fig 8I), and lactate production (Fig 8E), while SDH activity was not altered (Fig 7G) and SQR activity was increased in sphere cells (Fig 7H). CS activity was lower both in CD24^-^ cells and sphere cells (Fig 8F), and ATP levels were increased in CD24^-^ and decreased in sphere cells (Fig 8J). The fact that PGC1a was greatly increased in the sphere cells (Fig 8K) indicates increased mitochondrial biogenesis. Finally, we evaluated the different cell types for cell cycle distribution. While CD24^-^ cells showed no difference, sphere cells appear to be partially arrested in G1 (data not shown).

### Mitochondrially targeted vitamin E succinate efficiently kills mesospheres

MM is a very aggressive tumour with very few therapeutic options [34], which may be due to the presence of TICs in MMs; our results indicate high TIC-like component of MM cell populations (cf. Fig 1) and their increasing aggressiveness upon serial transplantations (cf. Fig 3). We therefore tested the susceptibility of mesospheres and Ist-Mes-2 adherent cells to several established anti-cancer agents, including doxorubicin 5-fluorouracil (5FU) and tamoxifen. We also tested α-tocopheryl succinate (α-TOS) that we found earlier efficient in killing MM cells [35] and its derivative, mitochondrially targeted vitamin E succinate (MitoVES), which we have reported to be some two orders more efficient in killing adherent MM cells than α-TOS [36]. Finally, we tested the effect of these agents on CD24 K/D Ist-Mes-2 cells. The IC_50_ values were significantly higher for sphere vs. adherent cells for doxorubicin and α-TOS, while no difference was found for 5FU and tamoxifen. However, the IC_50_ value was some 4-5-fold lower for sphere cells treated with MitoVES. Knocking down CD24 did not significantly affect the IC_50_ values for 5FU, tamoxifen and α-TOS. Adherent CD24^-^ cells showed IC_50_ values for MitoVES significantly higher than those for MitoVES-treated sphere cells (Table 2). These data clearly identify MitoVES as a compound considerably efficient in killing MM sphere cells and further support a role of CD24 in MM sphere formation.

**Table 2:** IC_50_ values for MitoVES and several other anti-cancer agents for parental and CD24^-^ adherent and sphere Ist-Mes-2 cells.

Anti-cancer agent	Adherent cells IC_50_	Sphere cells IC_50_	CD24^-^ cells IC_50_
Doxorubicin	14.79±1.23 μM	**20.74±3.45** μM^a^	N.D.
5FU	1.325±0.27 mM	1.325±0.27 mM	1.042±0.74 mM
Tamoxifen	27.95±0.3 μM	29.30±0.14 μM	29.85±0.11 μM
α-TOS	40.8±0.28 μM	**108.3±5.50** μM	42.33±0.28 μM
MitoVES	25.83±1.47 μM	**6.924±.0.5** μM	7.709±3.6 μM

^a^Numbers in bold indicate significantly different data (*p*<0.05) from adherent CD24^-^and parental cells.

The data are mean values ± S.D. from three independent experiments.

## Discussion

Malignant mesothelioma is one of the most aggressive tumours, is considerably resistant to current therapeutic modalities [3, 34, 37]. Novel approaches are necessary to identify the underlying mechanism(s) of resistance to improve therapeutic outcomes. The CSC (TIC) model has been shown to represent many features of cancer including drug resistance and metastasis. This concept was established based on similarities observed between normal stem cells and TICs. They both show extensive proliferation and the resultant tissue composed of various heterogeneous compartments with specific phenotypic characteristics [11]. Since the low response of MM to therapy may be attributed to the TIC subpopulation within the tumour, we investigated the existence and characteristics of cells with stem-like features in MM cell lines.

We based our study on previous observations indicating that anchorage-independent cells grown as spheres in SFM supplemented with growth factors are enriched with cells with increased ‘stemness’ and that such cells have the capacity to recapitulate the primary tumour when injected into immunocompromised mice [38]. Our data show that all MM cell lines tested can produce mesospheres with the ability to propagate and grow continuously in an anchorage-independent manner. This is indicative of the self-renewal property of mesospheres, a key feature of TICs. We confirmed this by showing that even a single MM cell is capable of forming spheres in SFM. We also conducted serial transplantation of mesospheres as a ‘gold standard’ of cancer cell self-renewal in immunodeficient mice [39].

The implications and advantages of formation of single cell-derived spheres were reviewed by Pastrana and colleagues [40]. The ability of a single cell to form proliferative spheres has another important biological implication. It can explain the notion that tumour formation is an inherent property of TICs rather than a ‘collaborative work’ of a group of cells to form the bulk of mesotheliomas. There are reports indicating that MM cells feature such a degree of plasticity, so that when transferred into proper environment, they can give rise to mesenchymal and other types of stem cells [41–43]. This notion is supported by our observation that, contrary to many other tumour types [44], the ratio of TICs to other malignant cells is very high in MM, and that a high number of MM cells have the propensity to ‘switch’ into TIC-like cells in the appropriate environment.

Besides increased overall level of stemness reported recently for Ist-Mes-2 spheres [27], mesospheres derived from all MM cell lines tested showed higher level of various stem cell markers, among which CD24, ABCG2, ABCB5 and OCT4 and also transcription factors SOX4, KLF4 and c-Myc are common to all of them. Importantly, regardless of the number of cells grafted (10^4^, 10^5^ and 10^6^), sphere cells formed tumours with a considerably higher rate than did their adherent counterparts, consistent with the known properties of TICs [10, 11, 16, 17, 45]. Also, sphere cell-derived tumours were lobular-like in appearance, indicating the possibility of several ‘tumour-initiating centres’.

Of considerable interest and, quite surprisingly, we found that upon serial transplantation, the sphere-derived tumours featured progressively shortened lag time, indicating that with each cycle of residence of the cells within the tumour microenvironment, the cells were ‘sculpted’ to better cope with the conditions, giving rise to progressively more aggressive tumour cells. Importantly, the individual generations of sphere cells also increased the level of the stemness markers CD24, ABCG2, OCT4 as well as EpCAM, and they also featured increased CS activity, indicative of alterations in the mitochondrial function. A change in the mitochondrial function in embryonic stem cells vs. their differentiated counterparts has been reported [46]. We further document this here for mesospheres by increased respiration of sphere cells derived from the ‘serial tumours’, which can be, to some extent, explained by progressively increased mitochondrial mass and cell size. We also observed slight increase in superoxide generation and enhanced glucose uptake and, to some extent, increased mitochondrial potential. TEM images from the individual generation sphere cells show considerable changes in mitochondrial morphology. These data document mitochondrial alterations in cells derived from individual tumour generations.

Since one of the important markers of MM stemness, CD24, progressively increased in the individual generations of tumour-derived spheres, in particular in those derived from Ist-Mes-2 cells), we tested whether it is important for MM stemness and for their efficacy to form tumours. We found that CD24^-^ cells lost their propensity to form spheres in SFM and that when grafted in NOD/SCID mice, their rate of tumour progression was >50% lower than that of their mock-transfected counterparts. The loss of sphere-forming capacity and efficacy of tumour formation for CD24^-^ MM cells can be linked to their loss of several other stemness markers, including CD47, EpCAM and OCT4, as shown for the tumour tissue. Although we cannot currently explain the potential causal link between CD24 and the other markers, it is obvious that there is a crosstalk of some kind between/among these individual genes and their products. Further, the lower proliferative capacity of CD24^-^ cells can be reconciled with lower level of pEGFR and pERK1/2, a component of the MAP kinase pathway, compared to parental and sphere cells. We also found that CD24 knock-down compromised respiration, whose capacity was, on the other hand, higher in sphere cells. While CD24^-^ cells did not considerably differ from parental cells in a variety of mitochondrial properties (except for lower CS activity and ΔΨ_m_, and higher ATP), the corresponding sphere cells showed significantly higher ΔΨ_m_, superoxide generation, lactate production and SQR activity, and showed lower ATP. Finally, CD24 knock-down did not considerably affect the cell cycle distribution, which revealed a G1 arrest for sphere cells. These findings document that mitochondrial function is altered upon CD24 suppression, the CD24 knock-down having an opposing effect than observed when parental cells were ‘converted’ to spheres.

Our data on the role of CD24 can be reconciled with a report documenting association of CD24 with poor outcome in mesothelioma patients [47], although additional work is needed to elucidate the exact mechanism(s) by which CD24 exerts its effect as a stem cell marker in MM and how it affects their stemness in general. A recent paper by Lee and colleagues documents that CD24 is important for self-renewal of liver TICs and their propensity to form tumours via a complex regulation involving STAT3 and NANOG [48].

Various studies were conducted to determine the mitochondrial and metabolic features of stem cells, and the results verify the fact that stem cells possess specific mitochondrial and metabolic features. It has been demonstrated that mitochondria in embryonic stem cells as well as induced pluripotent cells are less ‘developed’; they are present in lower numbers, are smaller and feature condensed morphology, contain less mtDNA and lower number of cristae with dilated morphology [49–53]. Metabolic activity of human pluripotent stem cells depends on glycolysis rather than OXPHOS. However, recent studies have shown that mitochondria of stem cells have functional OXPHOS machinery, which is uncoupled from respiration by proteins like UCP2 [54, 55]. Metabolic changes in pluripotent stem cells result in decreased levels of ATP and superoxide compared to more differentiated cells. However, higher levels of ROS in neural stem cells are related to active generation of ROS to control proliferation and survival [56]. Elevated ΔΨ_m,i_ has also been reported in embryonic stem cells and induced pluripotent stem cells [49, 57], which decreases upon differentiation. These findings can be extrapolated to MM TICs also exerting alterations in the mitochondrial function, as documented in this study.

Although there is considerable wealth of data describing the energetic features of normal stem cells, the metabolic and mitochondrial characteristics of TICs remain understudied with only one report pertaining to these aspects for lung TICs [58]. Considering similarities between stem cells and TICs, it is expected that lower levels of ATP and higher levels of lactate could be observed in the latter. Our data confirm that mesospheres produce less ATP compared to adherent cells. Another evidence of the glycolytic switch is a decrease of OXPHOS [46], once again rather consistent with our data on the relative O_2_ flux, indicating that cultured TICs utilise only a fraction of their ETC capacity.

We show that mesospheres are relatively resistant to several anti-cancer agents, including tamoxifen and doxorubicin. They can be inefficient due to the high level of ABCG2 that confers drug resistance [59] or because of the compromised p53 status of the cells [60]. We tested also α-TOS that induces selective apoptosis in malignant cells by acting via CII [61, 62]. While α-TOS was rather inefficient in killing MM sphere cells, MitoVES, derived by tagging the parental vitamin E succinate with a triphenylphosphonium group [63], was very efficient, killing >75% of mesosphere cells. IC_50_ values for sphere cells compared to adherent cells were either unchanged for 5FU and tamoxifen, or increased for doxorubicin and α-TOS. On the other hand, it was 4-5-fold lower for MitoVES. Furthermore, MitoVES killed CD24^-^ cells with IC_50_ value similar to that for mesosphere cells, consistent with the idea that CD24 also acts as a survival factor [64]. There are at least two reasons for the increased susceptibility of mesosphere cells to MitoVES compared to their adherent counterparts. MitoVES targets the UbQ site of CII [63], and mesospheres feature increased activity of SQR, which may result from increased level of CII. Further, MitoVES accumulates in mitochondria due to the delocalised cationic group TPP^+^. Importantly, mesosphere cells exert considerably higher ΔΨ_m_ than the adherent cells, and we have previously shown that dissipation of ΔΨ_m_ compromises mitochondrial uptake of MitoVES and its killing efficacy [63, 65].

In conclusion, we document that MM cell lines comprise a considerably large subset of TICs, using the anchorage-independent growth condition, and that the sphere cells are more efficient in tumour formation, in particular when grafted at low numbers, including their propensity to form tumours upon serial grafting with progressively increasing aggressiveness and increasing level of stemness, and this can be reconciled with their altered mitochondrial function. We further document that MM stemness is dependent on CD24, whose knock-down compromises the stem-like properties of the cells including efficient tumour progression. Finally, we show that mesospheres are susceptible to killing by MitoVES, consistent with their altered mitochondrial properties. Therefore, targeting mitochondria may be an efficient manner of combating TICs with translational consequences.

## Supporting Information

S1 Fig (TIF)
