# Correction: Characterisation of Mesothelioma-Initiating Cells and Their Susceptibility to Anti-Cancer Agents

**DOI:** 10.1371/journal.pone.0156012

**Published:** 2016-05-17

**Authors:** Elham Alizadeh Pasdar, Michael Smits, Michael Stapelberg, Martina Bajzikova, Marina Stantic, Jacob Goodwin, Bing Yan, Jan Stursa, Jaromira Kovarova, Karishma Sachaphibulkij, Ayenachew Bezawork-Geleta, Margaryta Sobol, Anatoly Filimonenko, Marco Tomasetti, Renata Zobalova, Pavel Hozak, Lan-Feng Dong, Jiri Neuzil

There is an error in the Funding section. The correct funding information is as follows: The work was supported in part by grants from the Australian Research Council, Cancer Council Queensland to J.N. and the Internal Grant Agency (IGA) of the Ministry of Health of the Czech Republic (IGA NT/14078-3), and the European Regional Development Fund (the BIOCEV project, CZ.1.05/1.1.00/02.0109) to JN. EAP was supported by the Douglas Francis Green PhD Scholarship provided by the Queensland Asbestos-Related Disease Society. LFD was supported by the Griffith University Research Fellowship. The funders had no role in study design, data collection and analysis, decision to publish, or preparation of the manuscript.
