# Supplementary figures and images for: Characterisation of Mesothelioma-Initiating Cells and Their Susceptibility to Anti-Cancer Agents
Source: PLoS One. 2015 May 1;10(5):e0119549. doi: 10.1371/journal.pone.0119549 (PMC4416766; doi:10.1371/journal.pone.0119549)

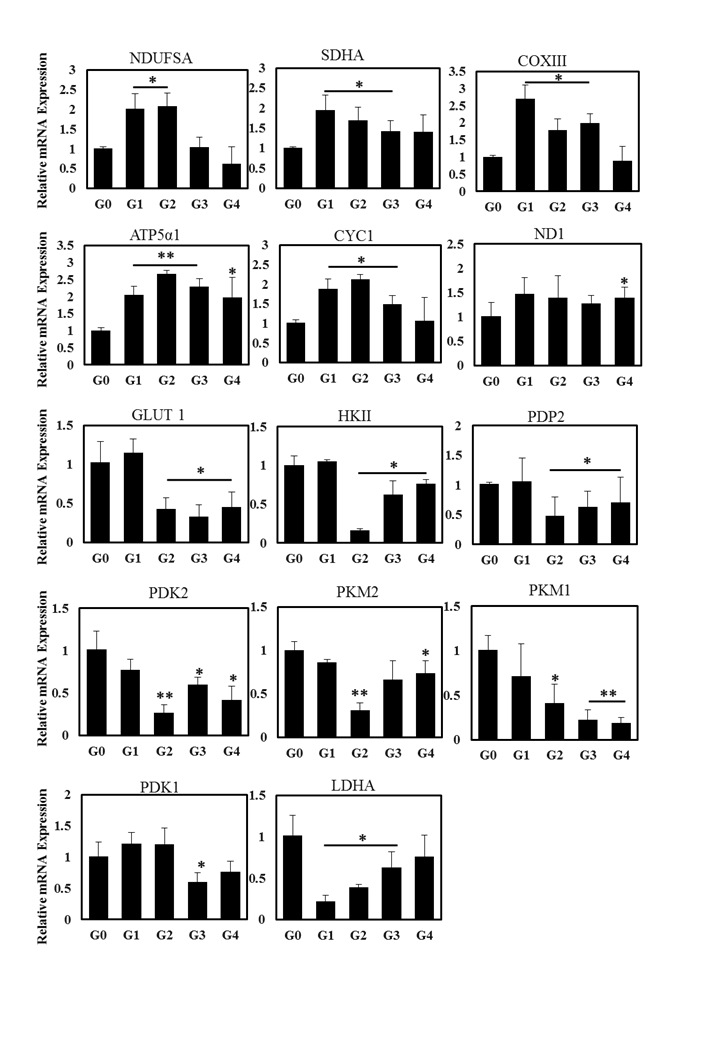

Supplement: S1 Fig — (TIF) [file pone.0119549.s001.tif]
